# Supplementary material for: The unfolded protein response reverses the effects of glucose on lifespan in chemically-sterilized C. elegans
Source: Nat Commun. 2022 Oct 19;13:5889. doi: 10.1038/s41467-022-33630-0 (PMC9582010; doi:10.1038/s41467-022-33630-0)
Supplement: Supplementary file 1 — Supplementary Information [file 41467_2022_33630_MOESM1_ESM.pdf]

SUPPLEMENTARY INFORMATION

The unfolded protein response reverses the effects of glucose on lifespan in chemically-sterilized *C. elegans*

Caroline BEAUDOIN-CHABOT, Lei WANG, Cenk CELIK, Aishah Tul-Firdaus ABDUL KHALID, Subhash THALAPPILLY, Shiyi XU, Jhee Hong KOH, Venus Wen Xuan LIM, Ann Don LOW, Guillaume THIBAULT

SUPPLEMENTARY FIGURES AND TABLES

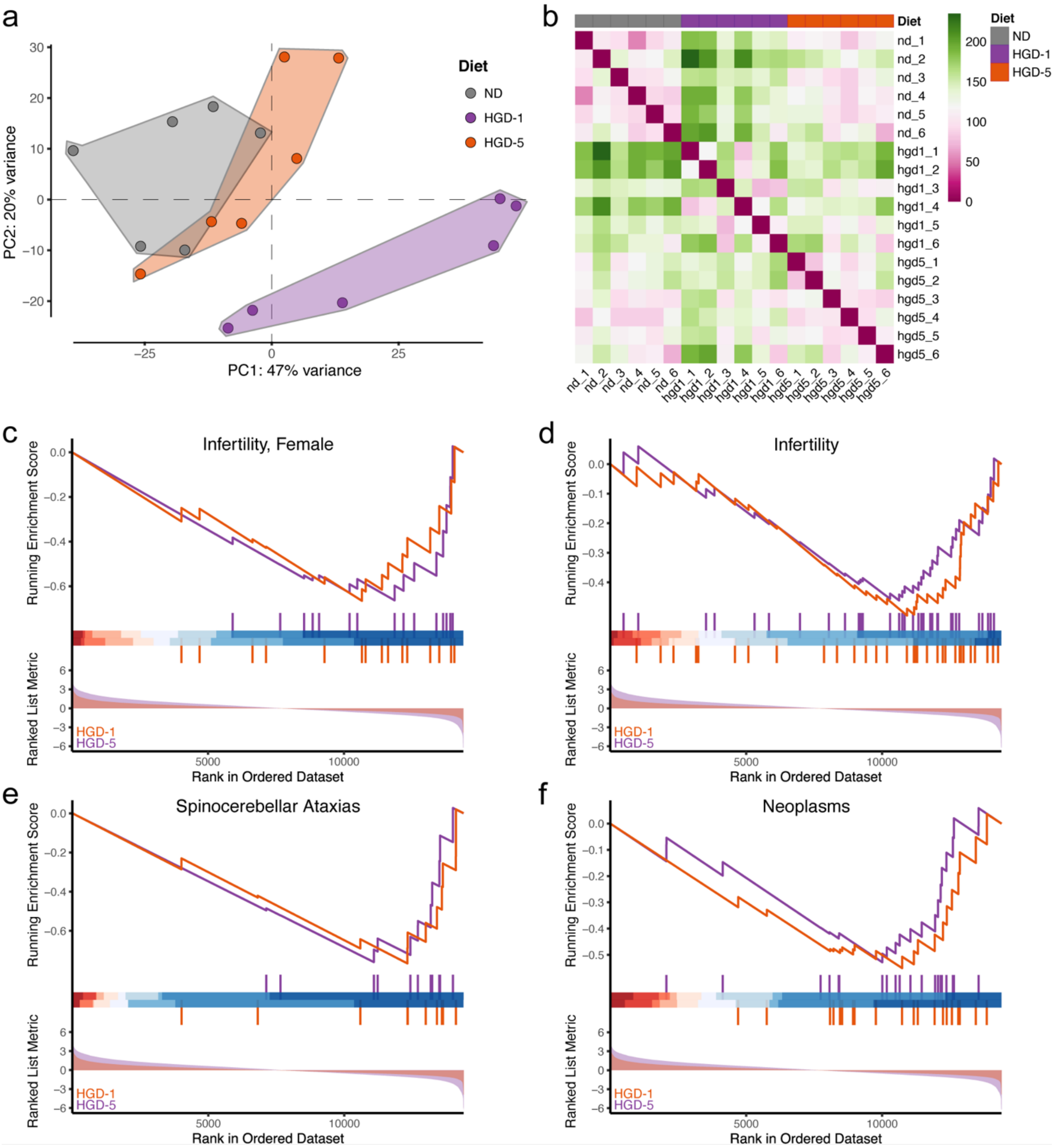

Supplementary Fig. 1 RNA-seq QC.

a PCA clusters of RNA-seq data and **b** the sample distances matrix for ND, HGD-1 and HGD-5. **c-f** Gene Set Enrichment Analyses comparing HGD-1 and HGD-5.

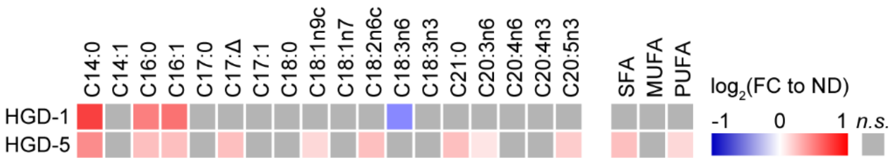

Supplementary Fig. 2 HGD induces fat storage in HGD-1 and HGD-5 animals.

Heat map of based 2 logarithmic fold changes (FC) in fatty acids (FAs), distribution of FA length, and saturation of 1- (HGD-1) and 5-day old (HGD-5) worms on 24h HGD compared to their respective WT ND (*n*=3). SFA, saturated fatty acid; MUFA, monounsaturated fatty acid; PUFA, polyunsaturated fatty acid. Two-tailed Student's t-test was performed to evaluate changes in each FA species. Grey boxes indicate non-significant changes in FA levels. P > 0.05.

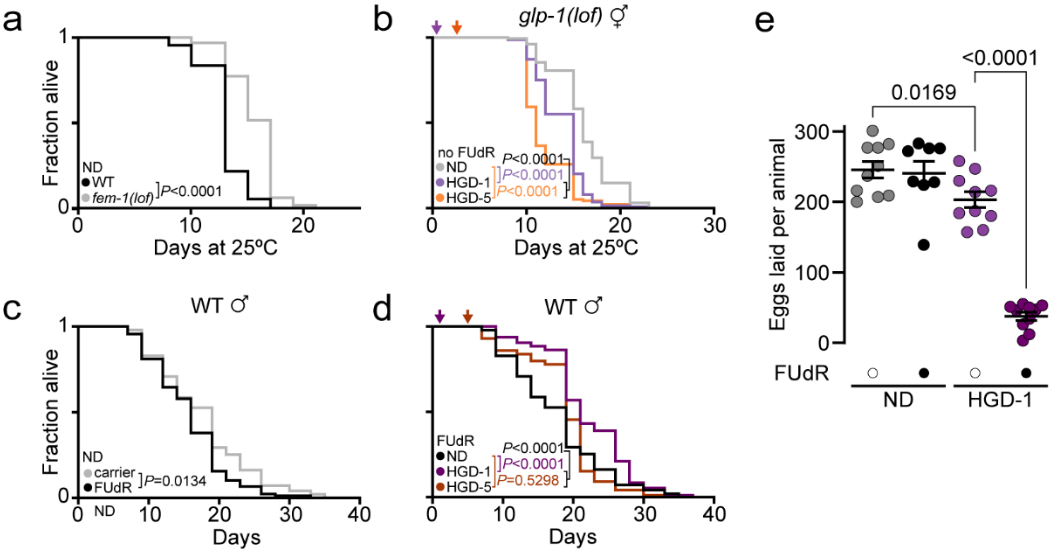

Supplementary Fig. 3 Lifespan assay of WT male animals.

a Lifespan assays of WT and *fem-1(lof)* hermaphrodite (WT, n=109; *fem-1*, n=92). **b** Lifespan assays of *glp-1(lof)* hermaphrodite (ND, n=242; HGD-1, n=248; HGD-5, n=236 including biological replicates) in the absence of 5-Fluoro-2'-deoxyuridine (FUdR). **c** Lifespan assays of WT male animals fed on ND in the absence or presence of FUdR (carrier, *n*=277; FUdR, *n*=307 including biological replicates). **d** Lifespan assays of WT hermaphrodite animals fed on ND, HGD-1, or HGD-5 in the presence of FUdR (ND, *n*=99; HGD-1, *n*=99; HGD-5, *n*=99). **e** Number of eggs laid per ND and HGD-1 day 4 animals in the absence (open circles) or presence (filled circles) of FUdR. Data shown are the mean ± SEM (*n*=10). Statistical analysis was subjected to log-rank test for lifespan (**a-d**) or one-way ANOVA with Tukey's test (**e**).

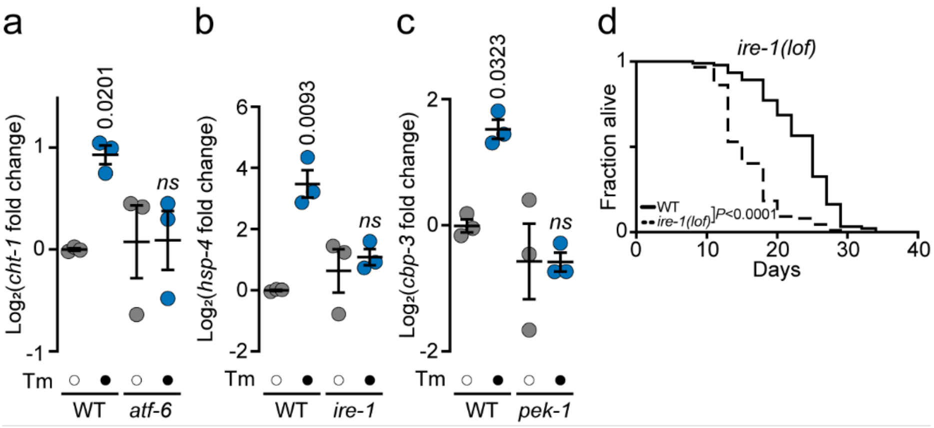

Supplementary Fig. 4 Validation of ER stress sensor target genes.

a qPCR comparing expression of genes *cht-1* in D1 WT and *atf-6(lof)* worms fed on the normal diet (ND) or ND followed by 4 h exposure to tunicamycin (Tm) (*n*=3). **b** qPCR comparing expression of genes *hsp-4* in D1 WT and *ire-1(lof)* worms treated as in **a** (*n*=3). **c** qPCR comparing expression of genes *F40F12.7* in D1 WT and *pek-1(lof)* worms treated as in **a** (*n*=3). *P* values to respective WT or mutant worms without Tm. *ns*, non-significant with *P*>0.05. **d** Lifespan assays of WT and *ire-1(lof)* animals fed on ND in the presence of FUdR (WT, *n*=100; *ire-1(lof)*, *n*=87). Data shown are the mean ± SEM. Statistical analysis was subjected to one-way ANOVA with Tukey's test (**a-c**) or log-rank test for lifespan (**d**).

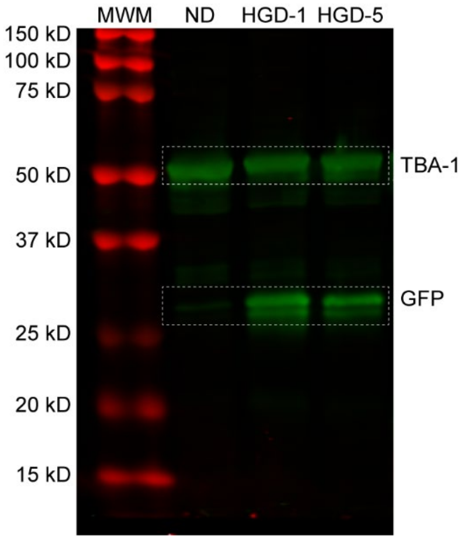

Supplementary Fig. 5 Representative blot of Fig. 3b.

Uncropped and unprocessed scan of the cropped blot in Fig. 3b.

Supplementary Data 1. Lifespan analysis.

Excel Spreadsheet

Supplementary Table 1. Oligonucleotide primers used in this study

Primer	Sequence (5' to 3')
ce_act-1 F	AGGACTGGGTGCTCTTCTGG
ce_act-1 R	GAGCACGGTATCGTCACCAA
ce_cbp-3 F	CATTGCCCGATGTGATCAGT
ce_cbp-3 R	CTGACATTGCCGAAGATCCAD11
ce_cht-1 F	TTGGGCTGGAGAAGGAATGT
ce_cht-1 R	ACTCCATCCTCCGAACGAGA
ce_hsp-4 F	CATCTCGTGGAATCAACCCT
ce_hsp-4 R	TGACGTCAAGAAGGACAACA
