# Supplementary material for: The unfolded protein response reverses the effects of glucose on lifespan in chemically-sterilized C. elegans
Source: Nat Commun. 2022 Oct 19;13:5889. doi: 10.1038/s41467-022-33630-0 (PMC9582010; doi:10.1038/s41467-022-33630-0)
Supplement: Supplementary file 2 — Reporting Summary [file 41467_2022_33630_MOESM2_ESM.pdf]

nature portfolio

Corresponding author(s): Guillaume Thibault

Last updated by author(s): Sep 3, 2022

Reporting Summary

Nature Portfolio wishes to improve the reproducibility of the work that we publish. This form provides structure for consistency and transparency in reporting. For further information on Nature Portfolio policies, see our Editorial Policies and the Editorial Policy Checklist.

Statistics

For all statistical analyses, confirm that the following items are present in the figure legend, table legend, main text, or Methods section.

n/a	Confirmed	
☐	☒	The exact sample size (*n*) for each experimental group/condition, given as a discrete number and unit of measurement
☐	☒	A statement on whether measurements were taken from distinct samples or whether the same sample was measured repeatedly
☐	☒	The statistical test(s) used AND whether they are one- or two-sided<br>*Only common tests should be described solely by name; describe more complex techniques in the Methods section.*
☐	☒	A description of all covariates tested
☐	☒	A description of any assumptions or corrections, such as tests of normality and adjustment for multiple comparisons
☐	☒	A full description of the statistical parameters including central tendency (e.g. means) or other basic estimates (e.g. regression coefficient) AND variation (e.g. standard deviation) or associated estimates of uncertainty (e.g. confidence intervals)
☐	☒	For null hypothesis testing, the test statistic (e.g. *F*, *t*, *r*) with confidence intervals, effect sizes, degrees of freedom and *P* value noted<br>*Give P values as exact values whenever suitable.*
☒	☐	For Bayesian analysis, information on the choice of priors and Markov chain Monte Carlo settings
☒	☐	For hierarchical and complex designs, identification of the appropriate level for tests and full reporting of outcomes
☒	☐	Estimates of effect sizes (e.g. Cohen's *d*, Pearson's *r*), indicating how they were calculated

Our web collection on statistics for biologists contains articles on many of the points above.

Software and code

Policy information about availability of computer code

Data collection	We used DinoCapture 2.0 to acquire videos of pharyngeal pumping, Tecan i-control software to quantify bacteria density, glucose and trehalose intake, Illumina HISEQ 4000 to acquire bulk RNA sequences, Odyssey CLx imaging system to acquire immunoblots, Zeiss Cell Observer to acquire neutral lipid stains, Shimadzu GC-2014 to acquire fatty acid levels, Zeiss LSM 800 confocal fluorescence microscope to acquire fluorescence microscope images, and CFX-96 Real-time PCR system to acquire quantitative real-time PCR.
Data analysis	We used Fiji software version 1.53c, GraphPad Prism version 9.2.0 for Windows, and RStudio version 4.0.4. Codes used in this study for quantitative bulk RNA sequencing are available at http://github.com/cenk-celik/c_elegans_rnaseq. R codes include Rsubread package (v2.4.3), DESeq2 (v1.30.1) , clusterProfiler (v4.0.5); meshes (v1.18.1), gage (v2.42.0), pathview (v1.32.0), pheatmap (v1.0.12) and ggplot2 (v3.3.5).

For manuscripts utilizing custom algorithms or software that are central to the research but not yet described in published literature, software must be made available to editors and reviewers. We strongly encourage code deposition in a community repository (e.g. GitHub). See the Nature Portfolio guidelines for submitting code & software for further information.

Data

Policy information about availability of data

All manuscripts must include a data availability statement. This statement should provide the following information, where applicable:

- Accession codes, unique identifiers, or web links for publicly available datasets
- A description of any restrictions on data availability
- For clinical datasets or third party data, please ensure that the statement adheres to our policy

Data availability
The source data for this study are available at the Dataverse Project doi.org/10.21979/N9/GEPEAG. All RNA sequencing data is available at the Gene Expression Omnibus accession number GSE182981.

Code availability
R codes used in this study for quantitative bulk RNA sequencing are available at http://github.com/cenk-celik/c_elegans_rnaseq.

Human research participants

Policy information about studies involving human research participants and Sex and Gender in Research.

Reporting on sex and gender	NA
Population characteristics	NA
Recruitment	NA
Ethics oversight	NA

Note that full information on the approval of the study protocol must also be provided in the manuscript.

Field-specific reporting

Please select the one below that is the best fit for your research. If you are not sure, read the appropriate sections before making your selection.

☒ Life sciences ☐ Behavioural & social sciences ☐ Ecological, evolutionary & environmental sciences

For a reference copy of the document with all sections, see nature.com/documents/nr-reporting-summary-flat.pdf

Life sciences study design

All studies must disclose on these points even when the disclosure is negative.

Sample size	Sample size was not predetermined using any statistical method. In our study, we followed standard protocols and procedures from the field. We used a sample size which is equal or above the average considered sufficient for each specific experimental setting.
Data exclusions	In general, data were excluded if the negative and positive controls failed to match expected results. Otherwise, data were not excluded.
Replication	Multiple trials and biological replicas of experiment were conducted. Number of biological replicates are indicated in the figure legends and were combined for data representation.
Randomization	Animals were randomly chosen for analysis. In each assay, worms were randomly selected for analysis from larger population growth under every experimental condition.
Blinding	The experimenters were not blinded. However, many of the experiments were conducted by different experimenters and shown to be consistent, including lifespan assays.

Reporting for specific materials, systems and methods

We require information from authors about some types of materials, experimental systems and methods used in many studies. Here, indicate whether each material, system or method listed is relevant to your study. If you are not sure if a list item applies to your research, read the appropriate section before selecting a response.

Materials & experimental systems

n/a	Involved in the study	
[ ]	[x]	Antibodies
[x]	[ ]	Eukaryotic cell lines
[x]	[ ]	Palaeontology and archaeology
[ ]	[x]	Animals and other organisms
[x]	[ ]	Clinical data
[x]	[ ]	Dual use research of concern

Methods

n/a	Involved in the study	
[x]	[ ]	ChIP-seq
[x]	[ ]	Flow cytometry
[x]	[ ]	MRI-based neuroimaging

Antibodies

Antibodies used	GFP (mouse anti-GFP; 1:1,000; Roche 11814460001) and IgG IRDye 800CW (anti-mouse IgG; 1:10,000; LiCOR Bioscences 925-32210).
Validation	The antibodies are commercially available and have been validated by their corresponding manufacturer's.

Animals and other research organisms

Policy information about studies involving animals; ARRIVE guidelines recommended for reporting animal research, and Sex and Gender in Research

Laboratory animals	C. elegans strains wild type N2, atf-6(ok551), daf-16(mu86), daf-16::GFP(tj356), fem-1(hc17), glp-1(e2144), ire-1(ok799), jnk-1(gk7), pek-1(ok275), rab-3p::xbp-1s(uthls270), xbp-1(zc12), far-3p::GFP(bc14852), and gly-19p::xbp-1s(uthls388) were used in this study.
Wild animals	No wild animals were used in this study.
Reporting on sex	NA
Field-collected samples	No field collected samples were used in the study.
Ethics oversight	No ethical approval is required to study Caenorhabditis elegans.

Note that full information on the approval of the study protocol must also be provided in the manuscript.
