# Supplementary material for: The unfolded protein response reverses the effects of glucose on lifespan in chemically-sterilized C. elegans
Source: Nat Commun. 2022 Oct 19;13:5889. doi: 10.1038/s41467-022-33630-0 (PMC9582010; doi:10.1038/s41467-022-33630-0)
Supplement: Supplementary file 4 — Description of Additional Supplementary Files [file 41467_2022_33630_MOESM4_ESM.pdf]

Title: Supplementary Data 1.
Description: Lifespan analysis. Lifespan analysis of different strains in the presence of absence of FUdR (5-fluorodeoxyuridine) treated with ND (normal diet); HGD-1, or HGD-5 (high glucose diet treatment at day 1 or 5 adult worms, respectively). L, indicates lifespan assays performed in liquid culture. **P values are compared to their respective treatments. ***bacteria were not UV-killed for these lifespans assay biological replicates. [#]Comparison of *ire-1(lof)* biological replicate on ND from Fig. 6f to WT on ND. Statistical analysis was subjected to log-rank test for lifespan.
